# A novel method for finding non-small cell lung cancer diagnosis biomarkers

**DOI:** 10.1186/1755-8794-6-S1-S11

**Published:** 2013-01-23

**Authors:** Quoc-Nam Tran

**Affiliations:** 1 Department of Computer Science, Lamar University, USA

## Abstract

**Background:**

One of the most common causes of worldwide cancer premature death is non-small cell lung carcinoma (NSCLC) with a very low survival rate of 8%-15%. Since patients with an early stage diagnosis can have up to four times the survival rate, discovering cost-effective biological markers that can be used to improve the diagnosis and prognosis of the disease is an important clinical challenge.

In the last few years, significant progress has been made to address this challenge with identified biomarkers ranging from 5-gene signatures to 133-gene signatures. However, A typical molecular sub-classification method for lung carcinomas would have a low predictive accuracy of 68%-71% because datasets of gene-expression profiles typically have tens of thousands of genes for just few hundreds of patients. This type of datasets create many technical challenges impacting the accuracy of the diagnostic prediction.

**Results:**

We discovered that a small set of nine gene-signatures (JAG1, MET, CDH5, ABCC3, DSP, ABCD3, PECAM1, MAPRE2 and PDF5) from the dataset of 12,600 gene-expression profiles of NSCLC acts like an inference basis for NSCLC lung carcinoma and hence can be used as genetic markers. This very small and previously unknown set of biological markers gives an almost perfect predictive accuracy (99.75%) for the diagnosis of the disease the sub-type of cancer. Furthermore, we present a novel method that finds genetic markers for sub-classification of NSCLC. We use generalized Lorenz curves and Gini ratios to overcome many challenges arose from datasets of gene-expression profiles. Our method discovers novel genetic changes that occur in lung tumors using gene-expression profiles.

**Conclusions:**

While proteins encoded by some of these gene-signatures (e.g., JAG1 and MAPRE2) have been showed to involve in the signal transduction of cells and proliferation control of normal cells, specific functions of proteins encoded by other gene-signatures have not yet been determined. Hence, this work opens new questions for structural and molecular biologists about the role of these gene-signatures for the disease.

## Background

Currently, cancer is a leading cause of death in the United States, second only to cardiovascular diseases. Each year, around 1.5 million people were diagnosed with cancer and more than half of a million people died from cancer, which makes cancer a major public health problem in the United States as well as many other parts of the world [1,2]. The top five most common cancer-related deaths were due to lung, breast, prostate, colorectal and pancreatic cancer. Together, these five diseases accounted for over 50% of all cancer deaths in the United States in 2009. Lung cancer alone, with NSCLC as the most common cause of worldwide cancer premature death, killed over 160,000 people, more than the other four cancers put together. The disease has a very low survival rate of 8%-15%. Meanwhile, the survival rate for patients with early-stage disease increases to 40%-55% after surgery. That said, discovering cost-effective biological markers that can be used to improve the diagnosis and prognosis of the disease is an important clinical challenge [3].

NSCLC is sub-categorized as adenocarcinomas, squamous cell carcinomas, and large-cell carcinomas, of which adenocarcinomas are the most common [4]. The histopathological sub-classification of lung adenocarcinoma is challenging. For example, in one study independent lung pathologists agreed on lung adenocarcinoma sub-classification in only 41% of cases [5]. In another study, proportional hazard models identified an optimal set of 50 prognostic mRNA transcripts using a 5-fold cross-validation procedure. This signature was tested in an independent set of 36 squamous cell lung carcinomas (SCC) samples and achieved 84% specificity and 41% sensitivity with an overall predictive accuracy of 68% [6]. Combining the SCC classifier with their adenocarcinoma prognostic signature gave a predictive accuracy of 71% in 72 NSCLC samples.

In the past few years, multiple techniques have evolved allowing rapid measurement of gene expression and simultaneous high-throughput measurement of thousands of genes from several hundred samples. Different parts of the gene-protein relationship can be measured such as messenger RNA levels, protein expression and cellular metabolic activity. Some of the available genomic technologies include gene expression arrays, serial analysis of gene expression, single-nucleotide polymorphism analysis, and high-throughput capillary sequencing [3]. Gene-expression array analysis methodologies developed over the last few years have demonstrated that expression data can be used in a variety of class discovery or class prediction biomedical problems including those relevant to tumor classification [7-10]. Data mining and statistical techniques applied to gene expression data have been used to address the questions of distinguishing tumor morphology, predicting post treatment outcome, and finding molecular markers for disease [11-14].

However, gene expression profiles present many challenges for data mining both in finding differentially expressed genes, and in building predictive models because the datasets are highly multidimensional (12,600 dimensions in our study) and contain a small number of records (197 records in our study). Although microarray analysis tool can be used as an initial step to extract most relevant features, one has to avoid over-fitting the data and deal with the very large number of dimensions of the datasets. The current challenges in analyzing gene-expression profiles, is illustrated in a method recently published in the Journal of Experimental & Clinical Cancer Research in July 2009 [15] where it used prior knowledge with support vector machine-based classification in diagnosis of lung cancer. The authors of [15] reported an accuracy of 98.51%-99.06% for their classification algorithm using 5 marker genes on a dataset of 31 malignant pleural mesothelioma (MPM) and 150 lung adenocarcinomas. Even though the method in [15] can differentiate between MPM and lung adenocarcinomas with high accuracy, it gives an accuracy of 70% when we added other types of NSCLC lung cancer including adenocarcinomas, squamous cell lung carcinomas and pulmonary carcinoids into consideration. Other researchers also limited themselves in differentiate two sub-types of NSCLC lung cancer such as between adenocarcinomas and squamous cell lung carcinomas.

This paper aims at a novel data mining method that finds cost-effective genetic markers and uses the markers to differentiate with very high accuracy all sub-types of NSCLC lung cancer. Comparing with recent publications in that the authors use currently available data mining techniques to find biomarkers for NSCLC lung cancer, we found that our new method finds significantly more cost-effective genetic markers and provides more accurate sub-classification of NSCLC lung cancer. Comparison with SAM [16], a popular method for significance analysis of microarrays, is also provided in this paper.

Our work is based upon the mRNA expression profiles in [17] in that a total of 203 snap-frozen lung tumors (n = 186) and normal lung (n = 17) specimens were used to create the dataset. Of these, 125 adenocarcinoma samples were associated with clinical data and with histological slides from adjacent sections. The 203 specimens include histologically defined lung adenocarcinomas (n = 139), squamous cell lung carcinomas (n = 21), pulmonary carcinoids (n = 20), and normal lung (n = 17) specimens. Total RNA extracted from samples was used to generate cRNA target, subsequently hybridized to human U95A oligonucleotide probe arrays according to standard protocols.

Among the nine gene-signatures found by our new method (JAG1, MET, CDH5, ABCC3, DSP, ABCD3, PECAM1, MAPRE2 and PDF5), proteins encoded by some of these gene-signatures (e.g., JAG1 and MAPRE2) have been showed to involve in the signal transduction of cells and proliferation control of normal cells [18]. It has also been found that MAPRE2 is highly expressed in pancreatic cancer cells, and seems to be involved in perineural invasion [19]. However, specific functions of proteins encoded by other gene-signatures have not yet been determined. Hence, this work opens new questions for structural and molecular biologists about the role of these gene-signatures for the disease.

## Results

### Finding genetic biomarkers

**Figure 1:**
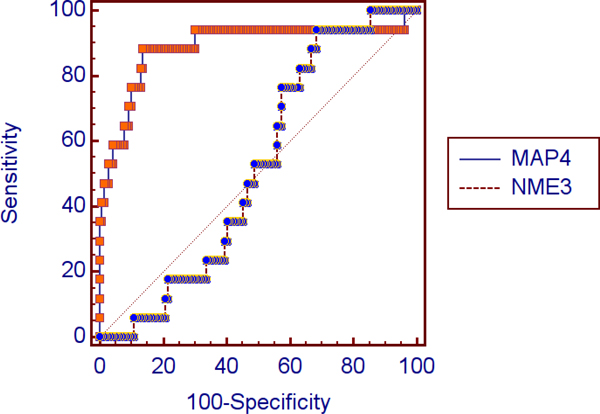
Comparing the ROC curves of a gene with high Lorenz-Gini value (MAP4) and a gene with low Lorenz-Gini value (NME3) for differentiating between lung adenocarcinomas and normal lung reveals the area under the curve, standard error and 95% confidence interval for MAP4 are 0.888, 0.0576 and 0.828-0.933 in comparison with 0.518, 0.0568 and 0.437-0.599 for NME3, respectively.

We first select 250 genes with the highest LorenzGini index values from a dataset of 12,600 gene-expression profiles for 197 patients using the novel algorithm described in Section Methods. Even though the genes with highest index values have some impact in differentiating the sub-types of NSCLC lung cancer (see Figure 1 for a statistical analysis), a simple use of these genes as biomarkers does not work because many of the genes are correlated and hence resulted in low overall accuracy for the predicting model. That said, one still has to look for good combinations of the high impact genes in order to find an accurate genetic biomarker subset.

To further reduce the size of the gene subsets and to improve the prediction accuracy, we evaluate different combinations of genes to identify an optimal subset in terms of accuracy for the Bayesian Network classification. Since it is infeasible to test all combinatorial possibilities from 250 genes, the gene subsets to be evaluated are generated using different subset search techniques. We use Best First and Greedy search methods in the forward and backward directions. Greedy search considers changes local to the current subset through the addition or removal of genes. For a given parent set, a greedy search examines all possible child subsets through either the addition or removal of genes. The child subset that shows the highest goodness measure then replaces the parent subset, and the process is repeated. The process terminates when no more improvement can be made. Best First search is similar to greedy search in that it creates new subsets based on the addition or removal of genes to the current subset with the ability to backtrack along the subset selection path to explore different possibilities when the current path no longer shows improvement. To prevent the search from backtracking through all possibilities in the gene space, a limit is placed on the number of non-improving subsets that are considered. In our evaluation we chose a limit of five.

The algorithm returns a set of nine genes (JAG1, MET, CDH5, ABCC3, DSP, ABCD3, PECAM1, MAPRE2 and PDF5) from the dataset of 12,600 gene-expression profiles of NSCLC. We exploit this small set of genes to differentiate all sub-types of NSCLC lung cancer.

**Figure 2:**
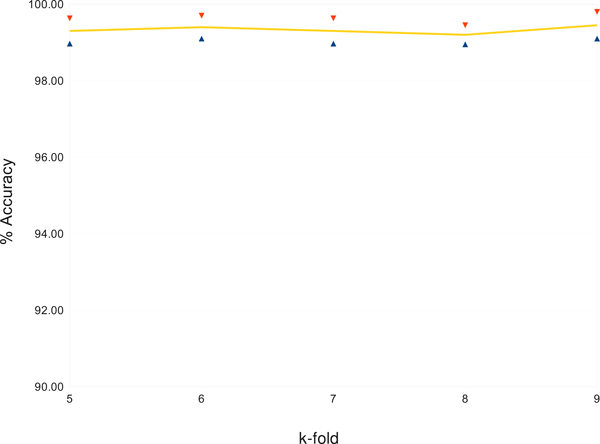
Accuracy of sub-classifications with standard deviations.

To build the classification model, we used Bayesian Network (BayesNet), which is explained in Section Methods. Figure 2 shows the averaged accuracies of the gene expression profile classification using Bayesian Network classification together with their standard deviations. To test the accuracy of classification models, we use *k*-fold cross validation, which is a common method for estimating the error of a model on benchmark medical data sets. For a reliable evaluation of the accuracy, we test the classification algorithm for many values of *k*. More precisely, we test for *k *= 5,6,7,8,9. For each value of *k*, the data set *D *is randomly divided into *k *subsets *D*_1_, *D*_2_, ..., *D_k_*. We leave out one of the subsets *D_i_*, *i *= 1..*k *each time for being used as a test data set for cross validation. The remaining subset ∪*_j≠i_D_j _*is used to build the model. The cross validation accuracy computed for each of the *k *test samples are then accumulated to give the *k*-fold estimate of the cross validation accuracy. To ease the effects of the random partitions on the data set, this whole process is repeated 50 times with different random seeds and the results are then averaged to give the estimated overall accuracy of the predicting model.

Notice that this testing approach separates the testing data from the training data when a model is built and hence avoiding the over-fitting situation. Furthermore, it allows us to have a total number of 197 gene-expression profiles as testing data.

During the validation process, all patients with lung adenocarcinomas were correctly predicted, all patients except one with squamous cell lung carcinomas were correctly predicted, all patients with pulmonary carcinoids were correctly predicted, and all patients with normal lung specimens were correctly predicted. The only false prediction for random seed 1 was a patient with squamous cell lung carcinomas but incorrectly predicted as adenocarcinomas. As we can see, this very small set of nine genes gives an almost perfect predictive accuracy for the diagnosis of the disease. When the number of genes is further reduced or increased, the accuracy starts to declined. That said, this set of nine genes acts like an inference basis for NSCLC lung carcinoma and hence can be used as genetic markers.

### Comparing with other gene selection methods

To investigate the classifying accuracy of the biomarkers generated by our new method, we first show that a method for molecular sub-classification is not a simple combination of binary classification models. Experimenting with binary classifications, we found that a biomarker singleton set of one gene, the STXBP1, provides 100% accuracy for differentiating between pulmonary carcinoids and normal lung. Similarly, a biomarker singleton set of one gene, the DOCK4, provides 100% accuracy for differentiating between squamous cell lung carcinomas and normal lung. A biomarker set of two genes, the MAP4 and SPP1, provides 99.36% accuracy for differentiating between lung adenocarcinomas and normal lung. However, combining these four genes together just give a molecular sub-classification model with 84.26% overall accuracy in comparison with 99.75% accuracy by our novel method.

**Figure 3:**
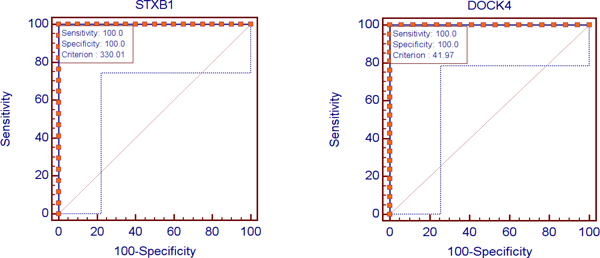
The ROC curves of STXB1 gene (left figure) for differentiating between pulmonary carcinoids and normal lung, and the ROC curves of DOCK4 gene (right figure) for differentiating between squamous cell lung carcinomas and normal lung.

Figure 3 shows the ROC curves of the genes STXBP1 and DOCK4.

Comparing with currently available data mining techniques in Weka to find biomarkers for NSCLC lung cancer, we found that our new method finds significantly more cost-effective genetic markers and provides more accurate sub-classification of NSCLC lung cancer. We also compare our method with SAM using the same dataset for NSCLC lung cancer. SAM combines t-test and permutations to calculate a False Discovery Rate to provide a subset of genes that are considered significant [16]. Using SAM, we select four sets of 50, 100, 150, 200 and 250 most significant genes by using the parameter values of 0.556. 0.458. 0.4188, 0.383 and 0.3568, respectively.

We then use the Bayesian Net classification in Weka to check the accuracy of the most significant gene sets generated by LorenzGini and SAM [20]. Besides our fresh implementation of LorenzGini algorithms, simple converters were written to connect SAM and Weka. For a reliable evaluation of the accuracy, we test the classification algorithm for many values of *k *as specified in our validation plan.

**Figure 4:**
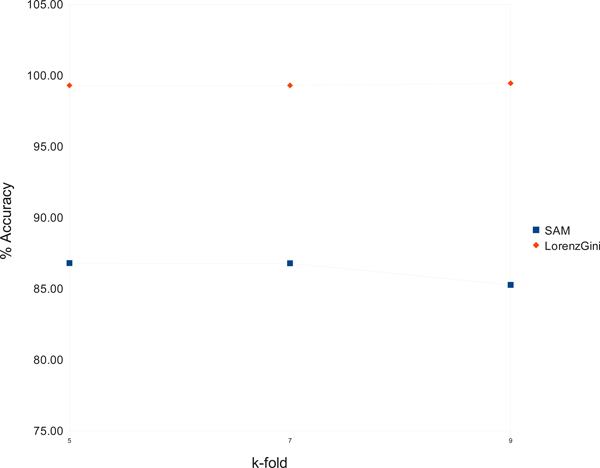
**SAM's & LorenzGini's gene sets classified by Bayesian Net**.

Figure 4 shows the accuracy of the gene expression profile classification using Bayesian Net algorithm on SAM's gene sets and on LorenzGini's gene sets with 50 genes. These two sets of 50 genes have only three genes in common: MEG3, CIRBP, and KCNK3. As we can see, the classifying accuracy has been improved with the LorenzGini's gene selections. We also observed that the accuracy of the gene expression profile classification using Bayesian Network algorithm on SAM's gene sets declined when the number of genes is reduced to 50 or smaller. In contrast, the accuracy of the gene expression profile classification using LorenzGini's gene sets is stable even when the number of genes is reduced to 9, which has the highest accuracy. This observation is also true for other classification methods.

## Conclusion

We presented a method that can find cost-effective biological markers as quantifiable measurements for an almost perfect predictive accuracy of NSCLC lung cancers. As cancers are complicated, one can only predict the status using a combination of many genes. The genes we discovered as genetic markers (JAG1, MET, CDH5, ABCC3, DSP, ABCD3, PECAM1, MAPRE2 and PDF5) are different with previously known results. Furthermore, proteins encoded by some of these gene-signatures (e.g., JAG1 and MAPRE2) have been showed to involve in the signal transduction of cells and proliferation control of normal cells while specific functions of proteins encoded by other gene-signatures have not yet been determined. Therefore, this work opens new questions for structural and molecular biologists about the role of these gene-signatures for the disease.

## Methods

An algorithm

**Input: **A gene-expression profiles dataset *D*

**Output: **A small subset of genes as genetic markers and a prediction model for NSCLC lung cancer

**Preparation: **Discretize the gene-expression profile values.

**Step1: **Pre-select 250 genes with highest ranking LorenzGini. (A threshold can be used for controlling the number of significant genes for genetic markers.)

**Step2: **Construct an optimal Bayesian network for a small set of genes as genetic markers that gives the highest overall accuracy for predicting all sub-type of NSCLC lung cancer.

In the subsequent subsections, we will provide the details for the steps of the algorithm

### Ranking the genes for biological markers

In order to find a small subset of genes as accurate biological markers from a gene-expression dataset with tens thousand of genes, one has to rank the genes with respect to some criteria. The criteria will be chosen so that the genes with highest index values have some impact in differentiating the sub-types of NSCLC lung cancer. However, current techniques in data mining such as the Gini index or the entropy approaches have limitation for this type of problem.

The first challenge that arose from the gene-expression datasets is the bias due to the order of cancer types or classes in data mining's terminology. Let's consider a simple example of expression profiles for a gene in Table 1 where the gene dataset *D *has *d *= 100 patients and three classes. The gene expression values were discretized into three ranges *R*_1_, *R*_2 _and *R*_3 _using for example the expectation-maximization method in [21]. Clearly, the cancer types or classes can be labeled in any order. When this gene is ranked by current microarray analysis methodologies, for example by calculating the Gini index $gini_{A}\left(D\right)={\text{Σ}}_{i=1}^{m}\frac{\left|R_{i}\right|}{d}\cdot gini\left(R_{i}\right)$, the first two rows contribute equally to the Gini index because $gini\left(R_{i}\right)=1-\sum_{j=1}^{n}p_{i,j}^{2}$ where $p_{i,j}=\frac{\left|C_{i,j}\right|}{\left|R_{i}\right|}$ is the relative frequency of class *C_j _*in *R_i_*, and |·| is the notation for cardinality [22]. We have the same problem when entropy is calculated instead of the Gini index. That said, when one just considers the probability distribution without taking into account the order of the classes, the first two rows will be considered the same. Clearly, the two rows should not be considered the same because row *R*_1 _says that 75% of patients with gene expression values within this range are classified into Class *C*_3 _while row *R*_2 _says that 75% of patients with gene expression values within this range are classified into Class *C*_2_. Hence, in order to have a robust gene selection method, one has to differentiate the partitions with different class orders because they have different amount of information.

**Table 1:** Bias due to the order of classes.

Range/Class	*C*_1_	*C*_2_	*C*_3_
*R*_1_	4	6	30
*R*_2_	6	30	4
*R*_3_	0	4	16

In this example, we consider the goodness ranking of a gene in a dataset with 100 patients and 3 classes of cancer sub-types. The expression values of the gene were discretized into 3 ranges *R*_1_, *R*_2 _and *R*_3_. There are 4 patients in class *C*_1 _with expression values in the range *R*_1_, 6 patients in class *C*_2 _with expression values in the range *R*_1_, and so on.

**Figure 5:**
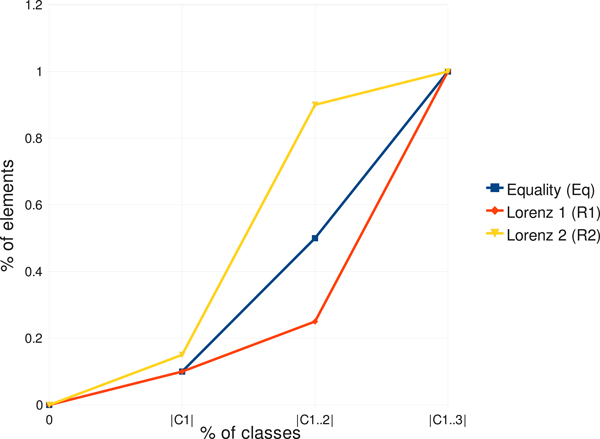
Lorenz curves.

To solve this problem, we generalized the well known Lorenz curves, a common measure in economics to gauge the inequalities in income and wealth. In Figure 5, we illustrate how modified Lorenz curves and modified Gini coefficients are calculated. The Equality Polygon (Eq) is defined based on the percentages of elements in $\left|C_{\text{1}}\right|,\;\left|C_{\text{1}..\text{2}}\right|\;=\;\left|C_{\text{1}}\right|\;+\;\left|C_{\text{2}}\right|,\;...,\;\left|C_{\text{1}..n}\right|\;=\;\sum_{j=1}^{n}\left|C_{j}\right|$ at *x*-coordinates 0, 1/*n*,2/*n*, ..., 1, where *n *is the number of classes and |*C*_1_| ≤ |*C*_2_| ≤, ..., ≤ |*C_n_*|. The Lorenz curve of a partition, say *R_i_*, is defined based on the percentage of elements in |*C_i_*,1|, $\left|{C_{i}}_{,\text{1}}\right|\;+\;\left|{C_{i}}_{,\text{2}}\right|,\;...,\;\sum_{j=1}^{n}\left|{C_{i}}_{,j}\right|$ at *x*-coordinates 0, 1/*n*,2/*n*, ..., 1. The Gini coefficient of a partition, say *R_i_*, is defined as $\left(\int_{0}^{1}L\left(R_{i}\right)\;\cdot \;dx-\int_{0}^{1}Eq\;\cdot \;dx\right)/\int_{0}^{1}Eq\cdot dx$. One can easily see that the partitions with different class orders are now differentiated. After being normalized, the coefficients can be used as weights in the calculation of the Gini index $gini_{A}\left(D\right)=\sum_{i=1}^{m}{\text{α}}_{i}\cdot \frac{\left|R_{i}\right|}{d}\cdot gini\left(R_{i}\right)$, where α*_i _*are the normalized coefficients.

Another technical challenge for microarray analysis methodologies comes from the order of discretized gene expression values. Let's consider another simple example of gene-expression profiles for two genes in Table 2 with three classes. The gene expression values were discretized into four ranges. In contrast to the previous challenge, the ranges of gene-expression values do follow some order. When this genes are ranked by current microarray analysis methodologies, for example by calculating the Gini index of gene *A *using dataset *D *$gini_{A}\left(D\right)=\sum_{i=1}^{m}\frac{\left|R_{i}\right|}{d}\cdot gini\left(R_{i}\right)$ where *d *= |*D*|, the two genes would have the same rank. Clearly, the gene-expression profiles on the right hand side of Table 2 have a more harmonic distribution with respect to the rows in comparison with the gene on the left. That said, these two genes should be ranked differently.

**Table 2:** Bias due to the order of gene expression values.

Class/Range	*C*_1_	*C*_2_	*C*_3_	Class/Range	*C*_1_	*C*_2_	*C*_3_
*R*_1_	3	0	0	*R*_1_	3	0	0
*R*_2_	0	88	0	*R*_2_	4	0	0
*R*_3_	4	0	0	*R*_3_	0	88	0
*R*_4_	0	0	5	*R*_4_	0	0	5

In this example, we consider the goodness ranking of two genes in a dataset with 100 patients and 3 classes of cancer sub-types. The expression values of the genes were discretized into 4 ranges *R*_1_, *R*_2_, *R*_3 _and *R*_4_. There are 4 patients in class *C*_1 _with expression values of gene #1 in the range *R*_3_, 88 patients in class *C*_2 _with expression values of gene #1 in the range *R*_2_, and so on.

To solve this problem, we generalized the Gini coefficients by taking into account the splitting status and the Gini ratio. The splitting status of *D *with respect to the attribute *A *is calculated as

$split_{A}\left(D\right)=1-\overset{m}{\underset{i=1}{\sum }}{\left(\frac{\left|R_{i}\right|}{d}\right)}^{2}.$

The Gini ratio of *D *with respect to the attribute *A *is defined as *LorenzGini*(*A*) = Δ*gini*(*A*)/*split_A_*(*D*), where $\text{Δ}gini\left(A\right)=gini\left(D\right)-gini_{A}\left(D\right)\text{ and }gini\left(D\right)=\text{1 }-\sum_{j=1}^{n}{\left(\frac{\left|C_{j}\right|}{d}\right)}^{2}$.

Furthermore, to take into account the gene expression profiles with different value orders, the Gini coefficient is calculated as $gini_{A}\left(D\right)=\sum_{i=1}^{m}\frac{\left|R_{i}\right|}{d}\cdot \text{δ}\left(i\right)\cdot gini\left(R_{i}\right)$, where *δ*(*i*) is the sum of the normalized distances between the row *i *and rows *i *- 1, *i *+1. The coefficient *δ*(*i*) is used as a weight to emphasize a row when it is close to its neighbors.

The splitting status of dataset *D *with respect to a gene can be calculated as a by-product when the reduction in impurity of *D *with respect to the gene is calculated. Therefore, the time complexity and space complexity of the algorithm are the same as the complexities of Gini index algorithm.

### Bayesian networks

After ranking the genes, one still has to look for good combinations of the high impact genes in order to find an accurate genetic biomarker subset because simply use the highest ranking genes as biomarkers does not work. The reason for this is that many of the highest ranking genes are correlated and hence resulted in low overall accuracy for the predicting model.

A Bayesian network (BN) is directed acyclic graph. The directed acyclic graph has a node for each of the genes and the class labels. Each node is associated with a color-coded table for the corresponding probability distribution related to the genes. In this example, we discretized the gene-expression profiles to simplify the tables. Each table has two parts. The left-hand side contains a column for each parent node. Each row on the right-hand side contains the probabilities that corresponds to one combination of values of the parents. To construct an optimal Bayesian network, we need a method to evaluate the goodness of a given network based upon the data and a method to search through the space of possible networks. We used the Akaike Information Criterion (AIC), which is the negation of the log-likelihood plus the number of parameters (i.e. 10 in this example) as a measuring score for evaluating the quality of a network. To search for an optimal network, we start with a given ordering of genes. We then process each node in turn and greedily consider adding edges that maximizes the network score. We also used other different searching strategies such as the Bayesian classification based method to compare the resulting networks. This searching method considers to add a second parent to each gene.

**Figure 6:**
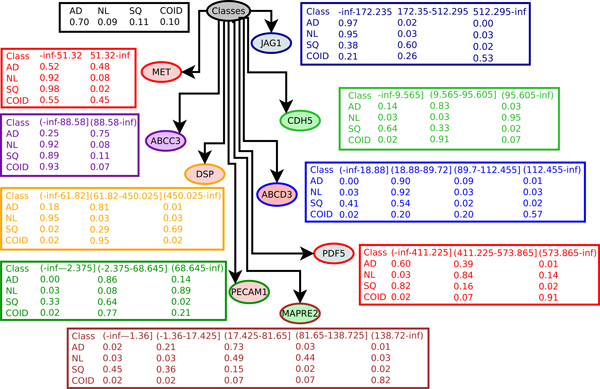
A Bayesian network (BN) predictive model for a 9 gene biomarker of JAG1, MET, CDH5, ABCC3, DSP, ABCD3, PECAM1, MAPRE2 and PDF5.

Once the predictive model is built as in Figure 6, we can use the model to predict whether a patient has NSCLC and the sub-type of cancer based upon the expression values of these nine genes. For instance, if the expression values of these nine genes are all zeros, the probabilities for this patient to be classified as normal is calculated as $\begin{array}{c} Pr[a_{JAG1}=0,a_{MET}=0,a_{CDH5}=0,a_{ABCC3}=0,a_{DSP}=0,a_{ABCD3}=0,a_{PECAM1}=0,a_{MAPRE2}=0,\;a_{PDF5}=0,\\ class=normal]=\prod_{i=1}^{9}Pr\left[a_{i}|{a_{i}}^{\prime }s\;\text{parents}\right]\cdot pr\left[class=normal\right]=0.95\cdot 0.92\cdot 0.03\cdot 0.92\cdot 0.95\cdot 0.03\cdot \\ 0.08\cdot 0.03\cdot 0.03\cdot 0.09=0.4454.10^{-8}. \end{array}$ Similarly, the probabilities for this patient to be classified as adenocarcinomas, squamous cell lung carcinomas and pulmonary carcinoids are 0, 0.3615 · 10^-4 ^and 0.2647 · 10^-10^, respectively. That said, this patient is determined as having squamous cell lung carcinomas.

Our method has been implemented in Maple, a C-like language, and Weka [20]. Notice that our new method works for any dataset with any number of classes. Even when the number of classes is equal to 2, the new method is completely different with other microarray analysis methodologies.

## Competing interests

The author declares that they have no competing interests.
